# Haste makes waste: Decision making in patients with restless legs syndrome with and without augmentation

**DOI:** 10.1371/journal.pone.0174793

**Published:** 2017-04-05

**Authors:** Beatrice Heim, Marie-Theres Pertl, Ambra Stefani, Margarete Delazer, Anna Heidbreder, Laura Zamarian, Elisabeth Brandauer, Klaus Seppi, Birgit Högl, Werner Poewe, Atbin Djamshidian

**Affiliations:** 1 Department of Neurology, Medical University of Innsbruck, Innsbruck, Tyrol, Austria; 2 Department of Molecular Neuroscience and Reta Lila Weston Institute for Neurological Studies, University of London, London, United Kingdom; Associazione OASI Maria SS, ITALY

## Abstract

**Objectives:**

To investigate decision making in patients with primary restless legs syndrome (RLS) with and without augmentation treated with dopaminergic medication.

**Methods:**

A total of 64 non-demented RLS patients treated with dopaminergic medication with and without augmentation were included in this study. We used an information sampling task to assess how much evidence participants gather before making a decision. Performance was compared to the results of 21 healthy controls.

**Results:**

All patients with and without augmentation gathered less information than healthy controls before making a decision (p<0.001), but there was no difference between the two patient groups (p = 1.0). Furthermore, both patient groups made more irrational decisions (e.g. decisions against the evidence they had at the time) than healthy controls (p≤0.002). In addition, RLS patients with augmentation made significantly more irrational decisions than RLS patients without augmentation (p = 0.037) and controls (p<0.001).

**Conclusions:**

Our results show that RLS patients treated with dopaminergic drugs, regardless of having augmentation or not, jumped to conclusions and decided significantly more often against the evidence they had at the time of their decision. However, those with augmentation performed worse than all other groups and made more often irrational decisions, a phenomenon which is also common in patients with substance abuse or behavioural addictions. Thus, jumping to conclusions and deciding with a higher degree of uncertainty as well as irrational decision making is more common in RLS patients treated with dopaminergic medication particularly in those with augmentation.

## Introduction

Restless legs syndrome (RLS) is a common sensorimotor disorder, which is frequently associated with neuropsychiatric comorbidities such as depression or panic disorder [1,2]. Dopaminergic drugs, especially non-ergot-derived dopamine agonists and alpha-2-delta ligands are considered as first-line therapies [3]. However, dopamine agonists may cause side effects such as impulse control disorders (ICDs) [4,5], augmentation or both [6–8] in a subgroup of treated patients.

Several studies have assessed various cognitive functions in RLS patients without augmentation. The results are inconsistent, with some studies showing decreased performance in tests measuring psychomotor speed, verbal fluency [9,10], decision making under ambiguity and reward seeking [11,12], while others even reported a superior performance on tasks assessing attention, language ability, executive function and memory [13,14], or no difference to healthy controls [15].

RLS patients with augmentation seem to score high on tests of psychological distress, compulsivity, depression and anxiety [16,17] and also exhibit often ICD symptoms [6]. It is, however, unclear whether these patients differ from RLS patients without augmentation on computerized neuropsychological tests assessing information sampling. Jumping to conclusions, without gathering sufficient information prior to making a decision, has been studied in detail in a large cohort of patients with and without addictive behaviours [18–22], but not in RLS patients so far.

Thus, the objective of the current study was to evaluate information sampling and decision making in treated RLS patients with and without augmentation. Given the previous literature [23] we hypothesized that both patients groups would jump to conclusions and make more irrational choices than healthy controls. Furthermore, we speculated that those with augmentation would perform significantly worse than all other groups, given that augmentation and impulse control disorders often coexist [6].

## Methods

The study was approved before initiation by the local Ethics Committee of the Medical University of Innsbruck, Austria, and all participants provided written informed consent according to the declaration of Helsinki.

### Subjects

We analysed information sampling in RLS patients treated with dopaminergic therapy with and without augmentation. Sixty-four RLS patients (40 with augmentation) were consecutively recruited from the sleep disorders outpatient clinic and sleep laboratory of the Medical University of Innsbruck, Department of Neurology. RLS severity was assessed using the International RLS study group rating scale (IRLS) [24]. Presence or absence of augmentation was assessed by RLS specialists at the sleep disorders outpatient clinic and sleep laboratory of the Medical University Innsbruck, Department of Neurology, according to current criteria [8] before they were included in the study. Furthermore, augmentation symptoms were categorized into mild and severe augmentation as recently proposed [3]. Results were compared to 21 healthy controls. Detailed medical and psychiatric assessments as well as relevant demographic characteristics were obtained (Table 1). Participants who scored less than 26/30 points on the Montreal Cognitive Assessment (MoCA) [25] as well as patients with major depression, a neurodegenerative disease, and those with untreated or therapy refractory internistic comorbidities (such as hypertension or asthma) were excluded. RLS patients without current dopaminergic treatment were not eligible for this study. Patients treated with a low dose of an antidepressant for mild depressive symptoms in the past and who were stable with their mood for the last 4 months were included (n = 4). The majority of the patients included here (n = 53) also participated in our study assessing the prevalence of ICD symptoms and augmentation in RLS [6]. Controls were recruited amongst patient’s spouses or family members. There were no differences in education, age and gender between the three groups. All participants performed a semi-structural interview to assess impulsive behaviour. Controls who screened positive for an impulse control disorder on the questionnaire for impulse control disorders (QUIP) [26] were excluded from the study.

**Table 1:** Demographic and clinical data of RLS patients (with and without augmentation) and healthy controls.

	RLS with Augmentation	RLS without Augmentation	Healthy Controls	p-Value
Number (n) • Mild augmentation • Severe augmentation	40 • 26 • 14	24	21	
ICDs • ICD symptoms ^b^ • Definite ICDs ^b^	19 • 6 • 13	3 • 3 • 0	0	0.006
Gender (male:female) ^b^	17:23	10:14	7:14	0.57
Age (years) ^a^	64.3 ± 10.0	58.6 ± 14.1	59.5 ± 11.1	0.147
Education (years) ^a^	10.5 ± 2.5	10.6 ± 2.6	11.0 ± 1.7	0.24
Disease duration (years) ^c^	18.0 ± 14.4	11.8 ± 11.4	NA	0.035
IRLS (at time of assessment) ^c^	24.7 ± 7.6	17.0 ± 7.8	NA	0.001
LEU (mg) ^c^	128.9 ± 155.6	36.1 ± 27.2	NA	0.002
Dopamine agonist monotherapy (all) • Pramipexole • Rotigotine • Ropinirole	29 • 23 • 4 • 2	21 • 16 • 3 • 2	NA	
Levodopa monotherapy	6	2	NA	
Levodopa plus dopamine agonists • Pramipexole • Rotigotine • Ropinirole	5 • 3 • 1 • 1	• 11	NA	

ICD = Impulse Control Disorder; IRLS = International Restless Legs Syndrome Study Group Rating Scale; LEU = Levodopa equivalent units; NA = not applicable. All values are mean ± SD.

^a^ Kruskal Wallis test.

^b^ Fisher’s Exact test.

^c^ Mann-Whitney U test.

### Beads task

All participants were tested on the Beads Task, which is a validated probabilistic reasoning task [27]. This task has been used in patients with Parkinson’s disease (PD) [20,23], schizophrenia [28], in patients with behavioural [19] and drug addictions [22] to assess reflection impulsivity, which is the amount of information gathered prior to making a decision. The test was performed in a quiet environment as described elsewhere [20].

In the Beads Task participants have to guess from which of two cups coloured beads are drawn. A blue cup contains mainly blue beads with fewer green ones and the green cup mainly green beads with fewer blue ones. The computer draws a single bead (blue or green) from either the blue or the green cup. Participants are shown the bead (blue or green) and are asked whether they want the computer to draw another bead from the same cup, or whether they want to guess immediately from which cup the bead was drawn. Participants are told that for every additional bead they let the computer draw, 0.2 points are deducted from their balance. They are also told that they can draw up to ten beads in each trial before making a decision. For every correct guess they win 10 points. Furthermore, participants are instructed that there are two different colour ratios of the beads present in the two cups. One is an 80:20 beads ratio where there are 80% blue beads and 20% green beads in the blue cup, and vice versa for the green cup; In the 60:40 condition 60% blue and 40% green beads are present in the blue cup and again vice versa for the green cup. Thus, participants are presented with two blocks (80:20; 60:40) consisting of three trials each and repeated the test once (in total six trials per ratio). This was done to assess whether there is a potential learning effect between first and second run. Prior to the test, subjects are shown a green and a blue cup containing coloured beads and perform a few practise trials to ensure that they all understand the task.

Relevant outcome measures in this task are number of draws prior to making a choice in each condition (‘drawing behaviour’) and number of decisions against the evidence (‘opposite colour choice’, e.g. blue bead shown, green cup chosen) collapsed over the four blocks.

The researchers (B.H. and A.D.) made sure that all participants understood the task. Drawn beads were placed next to the participant as poor working memory can also influence decision on information sampling tasks [29].

In addition, reaction times (RT) were recorded to assess how quickly participants made their decisions (draw a further bead or choose a cup).

### Statistics

Statistical analyses were performed using SPSS 22.0. The Fisher’s Exact test as well as parametric and non-parametric tests were used for statistical analysis depending on the distribution and the scale type of the variables. Data were analysed with parametric statistics where normality assumptions were met. Otherwise, non-parametric tests were used.

Drawing behaviour and irrational decision making (e.g. blue bead shown green cup selected) were calculated as described previously [20].

A generalized mixed linear model was used with either the number of draws before making a decision or the number of decisions that were contrary to the evidence participants had at the time (irrational decision making) as dependent variables (S1 File). A Poisson model which had a log-linear link function was used. For the analyses, beads ratio (80:20 or 60:40), condition (first/second run) and group (RLS-AUG, RLS+AUG versus controls) were modeled as fixed factors and subject was modeled as a random factor nested under group. A full model was fit, therefore each subject had individual coefficients for beads ratio, and group-level hypotheses were tested between subjects. All pairwise comparisons were Bonferroni corrected. A p-value below 0.05 (2-sided) was considered to indicate statistical significance.

## Results

Results of demographic and clinical variables of RLS patients with and without augmentation are shown in Table 1.

### Demographics

There was no age difference (χ^2^ = 2, p = 0.15) and no difference in years of education between the three groups (χ^2^ = 2, p = 0.24, Table 1). Of the 64 patients, 40 had clinical symptoms of augmentation, with 26 having mild and 14 severe augmentation according to recently published guidelines [30]. As expected, there were significant group differences between RLS patients with and without augmentation in Levodopa equivalent units (LEU) dose (U = 265.00, p = 0.002), International Restless Legs Study Group (IRLSSG) severity scale IRLS (U = 236.50, p = 0.001), and the presence of ICDs (χ^2^ = 8.145, exact p = 0.006) (Table 1). Furthermore, there was a significant difference in disease duration between RLS patients with and without augmentation (U = 328.50, p = 0.035). For further details see Table 1.

### Beads task

#### Drawing behaviour

**Fig 1:**
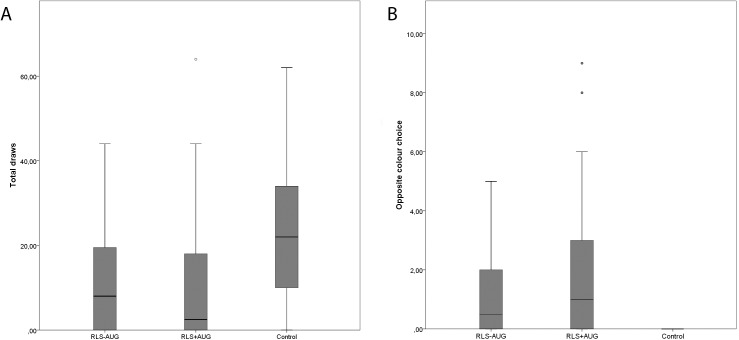
Active information sampling and irrational decisions compared between groups. Fig 1A. Drawing of beads and irrational decision making. Fig 1B. (RLS-AUG = RLS patients without augmentation; RLS+AUG = RLS patients with augmentation; HC = healthy controls). Box plot showing the median (horizontal line) within a box containing the central 50% of the observations (i.e., the upper and lower limits of the box are the 75^th^ and the 25^th^ percentiles) and extremes of the whiskers containing the central 95% of the ordered observations. Outliners are shown as circles.

There was a significant effect of group (RLS patients with and without augmentation, HC) (Wald χ^2^ = 61.39, p<0.001), beads ratio (60:40; 80:20) (Wald χ^2^ = 130.13, p<0.001) as well as a significant interaction between group and beads ratio (Wald χ^2^ = 24.19, p<0.001). There was no effect of condition (first/second run) (Wald χ^2^ = 0.1, p = 0.72) and no interaction of group and condition (Wald χ^2^ = 2.0, p = 0.36). In total, RLS patients with augmentation drew 11.68 (95% CI 10.66; 12.78), RLS patients without augmentation drew 11.25 (95% CI 9.99; 12.68) beads. In contrast, healthy controls picked 22.86 (95% CI 20.90; 25.00) beads before making a decision. Pairwise comparisons revealed that both RLS groups gathered significantly less information than healthy volunteers (both p-values<0.001) but there was no difference between RLS patients with and without augmentation (p = 1.0) (Fig 1A). As there were significant differences in LEU dose, IRLS and disease duration between the two RLS patient groups, we have added these three demographic characteristics as a covariate in a multivariate analysis. The results remained unchanged. There was no difference between RLS patients with and without augmentation in drawing behaviour (Wald χ^2^ = 0.04, p = 0.96).

Furthermore, all participants drew more beads in the high conflict (60:40) compared to the low conflict (80:20) condition. Moreover, in the 60:40 condition there was a significant effect of group (Wald χ^2^ = 41.7, p<0.001). Pairwise comparisons showed that both RLS patient groups drew significantly fewer beads than HC (p≤0.03). There was no difference between the two RLS groups. In the 80/20 condition we found a significant group difference (Wald χ^2^ = 6.05, p = 0.038), but pairwise comparisons did not reach significance (all p-values >0.1).

#### Irrational decision making/opposite colour choice

There was a significant effect of group in irrational decision making (Wald χ^2^ = 23.48, p<0.001). In total, RLS patients with augmentation chose 2.00 (95% CI 0.84; 2.49), RLS patients without augmentation 1.21 (95% CI 0.84; 1.74), and healthy controls 0.33 (95% CI 0.16; 0.70) times against the evidence. Post hoc analyses showed that RLS patients with (p<0.001) and without augmentation (p = 0.002) made significantly more irrational decisions than healthy volunteers. Furthermore, RLS patients with augmentation made significantly more irrational decisions than RLS patients without augmentation (p = 0.037) (Fig 1B). Again we have performed a multivariate model to account for differences in LEU dose, IRLS and disease duration. The result remained, however, unchanged. RLS patients with augmentation made significantly more irrational decisions than RLS patients without augmentation (Wald χ^2^ = 7.9, p = 0.012).

## Discussion

This is the first study to assess jumping to conclusion behaviour in RLS patients with and without augmentation. We found that both patient groups gathered significantly less information and made more irrational decisions, e.g. deciding against the evidence they had at the time of their decisions, than healthy controls.

Furthermore, RLS patients with augmentation also decided significantly more often against the evidence than RLS patients without augmentation. This is particularly interesting as the majority of RLS patients with augmentation had only mild augmentation symptoms (65%). Whether these irrational decisions are a result of the higher dopaminergic dose, a disease specific neurocognitive change, or an intrinsic impulsive personality trait that contributes to the development of augmentation, remains unclear.

Sampling less information before making a decision, or “jumping to conclusions”, as well as irrational decision making is common in drug naïve PD patients [21], PD patients with impulse control disorders [20], patients with schizophrenia [31], binge drinkers [19], and substance abusers [18,22]. A neuroimaging study in healthy controls showed that a wide brain network including the parietal and prefrontal cortex, the anterior insula, and also the striatum are activated during the beads task [32].

It is, however, unclear whether treated RLS patients simply gather less information (e.g. have a lower decision threshold), overvalue the evidence they have already (e.g. overconfidence) or a combination of both factors [31]. It is likely that dopamine agonists contribute to the impairment in decision making on the beads task. Dopamine agonists are known to contribute to jumping to conclusion behaviour in PD [23], impulsive choice, temporal discounting [33] and delusional thinking, which has been linked with poor performance on the beads task [31,34]. Impairment of information sampling, regardless of whether it is due to overconfidence or a lower decision threshold, is possibly caused by reduced “top down” cortical inhibition. In line with this hypothesis dopamine agonists are known to reduce prefrontal cortex activation [35]. Furthermore, two studies have shown that dysfunction of the prefrontal cortex [36] as well as a smaller prefrontal cortex [19] are associated with jumping to conclusion behaviour. A decrease in grey matter volume in the parietal lobes, medial frontal areas and lateral temporal areas has been demonstrated in RLS [37]. However, whether these changes are indeed responsible for reflection impulsivity in RLS is still unclear.

Our results are in line with previous neuropsychological studies in RLS demonstrating increased risk taking under ambiguity and reduced learning from long term negative consequences [11,12].The findings presented here expand the current literature as we show that RLS patients gather less information than healthy controls, take premature decisions and often make irrational choices. Moreover, the present study indicates that RLS patients with augmentation have more pronounced irrational reasoning than RLS patients without augmentation.

However, we need to highlight potential limitations of our study. As RLS patients with augmentation scored higher on the IRLS, it is possible that sleep deprivation may contribute to poorer task performance, although the relationship of sleep deprivation and decision making is far from established [13,38] and we did not specifically assess sleep deprivation and quality of sleep.

## Conclusions

We found that both RLS patient groups jumped to conclusions and made more decisions against the evidence on the beads task than healthy controls. Furthermore, patients with augmentation made significantly more often irrational decisions than controls and RLS patients without augmentation. This behaviour is often seen in patients with substance abuse [18,20], patients with schizophrenia [31] and those with behavioural addictions [20,39]. Whether this trait is also found in RLS patients treated with alpha-2-delta ligands needs to be explored in future studies.

## Supporting information

S1 File Beads task performance. Drawing behaviour of all participants.
(DOCX)
