# Supplementary material for: Haste makes waste: Decision making in patients with restless legs syndrome with and without augmentation
Source: PLoS One. 2017 Apr 5;12(4):e0174793. doi: 10.1371/journal.pone.0174793 (PMC5381880; doi:10.1371/journal.pone.0174793)
Supplement: S1 File — Drawing behaviour of all participants. (DOCX) [file pone.0174793.s001.docx]

| **Subject** | **Ratio** | **Draws** | **Group Condition** | |  |  |  |  |  |
| --- | --- | --- | --- | --- | --- | --- | --- | --- | --- |
| 1 | 80 | 0 | RLS | 1 |  |  |  |  |  |
| 1 | 80 | 2 | RLS | 2 |  |  |  |  |  |
| 1 | 60 | 3 | RLS | 1 |  |  |  |  |  |
| 1 | 60 | 4 | RLS | 2 |  |  |  |  |  |
| 2 | 80 | 0 | RLS | 1 |  |  |  |  |  |
| 2 | 80 | 0 | RLS | 2 |  |  |  |  |  |
| 2 | 60 | 5 | RLS | 1 |  |  |  |  |  |
| 2 | 60 | 11 | RLS | 2 |  |  |  |  |  |
| 3 | 60 | 13 | RLS | 1 |  |  |  |  |  |
| 3 | 80 | 12 | RLS | 2 |  |  |  |  |  |
| 3 | 80 | 12 | RLS | 1 |  |  |  |  |  |
| 3 | 60 | 7 | RLS | 2 |  |  |  |  |  |
| 4 | 80 | 0 | RLS+AUG | 1 |  |  |  |  |  |
| 4 | 80 | 3 | RLS+AUG | 2 |  |  |  |  |  |
| 4 | 60 | 3 | RLS+AUG | 1 |  |  |  |  |  |
| 4 | 60 | 0 | RLS+AUG | 2 |  |  |  |  |  |
| 5 | 80 | 0 | RLS+AUG | 1 |  |  |  |  |  |
| 5 | 80 | 0 | RLS+AUG | 2 |  |  |  |  |  |
| 5 | 60 | 0 | RLS+AUG | 1 |  |  |  |  |  |
| 5 | 60 | 0 | RLS+AUG | 2 |  |  |  |  |  |
| 6 | 80 | 0 | RLS+AUG | 1 |  |  |  |  |  |
| 6 | 80 | 0 | RLS+AUG | 2 |  |  |  |  |  |
| 6 | 60 | 0 | RLS+AUG | 1 |  |  |  |  |  |
| 6 | 60 | 0 | RLS+AUG | 2 |  |  |  |  |  |
| 8 | 80 | 6 | RLS+AUG | 1 |  |  |  |  |  |
| 8 | 80 | 8 | RLS+AUG | 2 |  |  |  |  |  |
| 8 | 60 | 15 | RLS+AUG | 1 |  |  |  |  |  |
| 8 | 60 | 15 | RLS+AUG | 2 |  |  |  |  |  |
| 9 | 80 | 0 | RLS+AUG | 1 |  |  |  |  |  |
| 9 | 80 | 0 | RLS+AUG | 2 |  |  |  |  |  |
| 9 | 60 | 0 | RLS+AUG | 1 |  |  |  |  |  |
| 9 | 60 | 0 | RLS+AUG | 2 |  |  |  |  |  |
| 10 | 80 | 4 | RLS+AUG | 1 |  |  |  |  |  |
| 10 | 80 | 10 | RLS+AUG | 2 |  |  |  |  |  |
| 10 | 60 | 23 | RLS+AUG | 1 |  |  |  |  |  |
| 10 | 60 | 27 | RLS+AUG | 2 |  |  |  |  |  |
| 11 | 80 | 1 | RLS+AUG | 1 |  |  |  |  |  |
| 11 | 80 | 0 | RLS+AUG | 2 |  |  |  |  |  |
| 11 | 60 | 4 | RLS+AUG | 1 |  |  |  |  |  |
| 11 | 60 | 3 | RLS+AUG | 2 |  |  |  |  |  |
| 12 | 80 | 0 | RLS+AUG | 1 |  |  |  |  |  |
| 12 | 80 | 2 | RLS+AUG | 2 |  |  |  |  |  |
| 12 | 60 | 5 | RLS+AUG | 1 |  |  |  |  |  |
| 12 | 60 | 0 | RLS+AUG | 2 |  |  |  |  |  |
| 14 | 80 | 0 | RLS+AUG | 1 |  |  |  |  |  |
| 14 | 80 | 0 | RLS+AUG | 2 |  |  |  |  |  |
| 14 | 60 | 1 | RLS+AUG | 1 |  |  |  |  |  |
| 14 | 60 | 0 | RLS+AUG | 2 |  |  |  |  |  |
| 15 | 80 | 0 | RLS+AUG | 1 |  |  |  |  |  |
| 15 | 80 | 0 | RLS+AUG | 2 |  |  |  |  |  |
| 15 | 60 | 3 | RLS+AUG | 1 |  |  |  |  |  |
| 15 | 60 | 1 | RLS+AUG | 2 |  |  |  |  |  |
| 16 | 80 | 4 | RLS | 1 |  |  |  |  |  |
| 16 | 80 | 5 | RLS | 2 |  |  |  |  |  |
| 16 | 60 | 6 | RLS | 1 |  |  |  |  |  |
| 16 | 60 | 5 | RLS | 2 |  |  |  |  |  |
| 17 | 80 | 0 | RLS+AUG | 1 |  |  |  |  |  |
| 17 | 80 | 0 | RLS+AUG | 2 |  |  |  |  |  |
| 17 | 60 | 4 | RLS+AUG | 1 |  |  |  |  |  |
| 17 | 60 | 6 | RLS+AUG | 2 |  |  |  |  |  |
| 18 | 80 | 4 | RLS | 1 |  |  |  |  |  |
| 18 | 80 | 5 | RLS | 2 |  |  |  |  |  |
| 18 | 60 | 6 | RLS | 1 |  |  |  |  |  |
| 18 | 60 | 4 | RLS | 2 |  |  |  |  |  |
| 20 | 80 | 0 | RLS+AUG | 1 |  |  |  |  |  |
| 20 | 80 | 0 | RLS+AUG | 2 |  |  |  |  |  |
| 20 | 60 | 5 | RLS+AUG | 1 |  |  |  |  |  |
| 20 | 60 | 12 | RLS+AUG | 2 |  |  |  |  |  |
| 22 | 80 | 0 | RLS | 1 |  |  |  |  |  |
| 22 | 80 | 0 | RLS | 2 |  |  |  |  |  |
| 22 | 60 | 0 | RLS | 1 |  |  |  |  |  |
| 22 | 60 | 0 | RLS | 2 |  |  |  |  |  |
| 23 | 80 | 4 | RLS | 1 |  |  |  |  |  |
| 23 | 80 | 5 | RLS | 2 |  |  |  |  |  |
| 23 | 60 | 6 | RLS | 1 |  |  |  |  |  |
| 23 | 60 | 5 | RLS | 2 |  |  |  |  |  |
| 24 | 80 | 5 | RLS+AUG | 1 |  |  |  |  |  |
| 24 | 80 | 8 | RLS+AUG | 2 |  |  |  |  |  |
| 24 | 60 | 12 | RLS+AUG | 1 |  |  |  |  |  |
| 24 | 60 | 8 | RLS+AUG | 2 |  |  |  |  |  |
| 26 | 80 | 0 | RLS | 1 |  |  |  |  |  |
| 26 | 80 | 0 | RLS | 2 |  |  |  |  |  |
| 26 | 60 | 0 | RLS | 1 |  |  |  |  |  |
| 26 | 60 | 0 | RLS | 2 |  |  |  |  |  |
| 27 | 80 | 0 | RLS+AUG | 1 |  |  |  |  |  |
| 27 | 80 | 0 | RLS+AUG | 2 |  |  |  |  |  |
| 27 | 60 | 0 | RLS+AUG | 1 |  |  |  |  |  |
| 27 | 60 | 0 | RLS+AUG | 2 |  |  |  |  |  |
| 30 | 80 | 0 | RLS | 1 |  |  |  |  |  |
| 30 | 80 | 0 | RLS | 2 |  |  |  |  |  |
| 30 | 60 | 0 | RLS | 1 |  |  |  |  |  |
| 30 | 60 | 0 | RLS | 2 |  |  |  |  |  |
| 31 | 80 | 0 | RLS | 1 |  |  |  |  |  |
| 31 | 80 | 5 | RLS | 2 |  |  |  |  |  |
| 31 | 60 | 7 | RLS | 1 |  |  |  |  |  |
| 31 | 60 | 0 | RLS | 2 |  |  |  |  |  |
| 32 | 80 | 0 | RLS+AUG | 1 |  |  |  |  |  |
| 32 | 80 | 0 | RLS+AUG | 2 |  |  |  |  |  |
| 32 | 60 | 0 | RLS+AUG | 1 |  |  |  |  |  |
| 32 | 60 | 0 | RLS+AUG | 2 |  |  |  |  |  |
| 34 | 80 | 5 | RLS+AUG | 1 |  |  |  |  |  |
| 34 | 80 | 4 | RLS+AUG | 2 |  |  |  |  |  |
| 34 | 60 | 11 | RLS+AUG | 1 |  |  |  |  |  |
| 34 | 60 | 7 | RLS+AUG | 2 |  |  |  |  |  |
| 35 | 80 | 0 | RLS | 1 |  |  |  |  |  |
| 35 | 80 | 0 | RLS | 2 |  |  |  |  |  |
| 35 | 60 | 4 | RLS | 1 |  |  |  |  |  |
| 35 | 60 | 3 | RLS | 2 |  |  |  |  |  |
| 36 | 60 | 0 | RLS+AUG | 1 |  |  |  |  |  |
| 36 | 80 | 0 | RLS+AUG | 2 |  |  |  |  |  |
| 36 | 60 | 0 | RLS+AUG | 1 |  |  |  |  |  |
| 36 | 80 | 0 | RLS+AUG | 2 |  |  |  |  |  |
| 37 | 80 | 6 | RLS+AUG | 1 |  |  |  |  |  |
| 37 | 80 | 7 | RLS+AUG | 2 |  |  |  |  |  |
| 37 | 60 | 11 | RLS+AUG | 1 |  |  |  |  |  |
| 37 | 60 | 16 | RLS+AUG | 2 |  |  |  |  |  |
| 38 | 80 | 4 | RLS | 1 |  |  |  |  |  |
| 38 | 80 | 5 | RLS | 2 |  |  |  |  |  |
| 38 | 60 | 9 | RLS | 1 |  |  |  |  |  |
| 38 | 60 | 0 | RLS | 2 |  |  |  |  |  |
| 39 | 80 | 0 | RLS | 1 |  |  |  |  |  |
| 39 | 80 | 0 | RLS | 2 |  |  |  |  |  |
| 39 | 60 | 0 | RLS | 1 |  |  |  |  |  |
| 39 | 60 | 0 | RLS | 2 |  |  |  |  |  |
| 40 | 80 | 0 | RLS+AUG | 1 |  |  |  |  |  |
| 40 | 80 | 2 | RLS+AUG | 2 |  |  |  |  |  |
| 40 | 60 | 12 | RLS+AUG | 1 |  |  |  |  |  |
| 40 | 60 | 21 | RLS+AUG | 2 |  |  |  |  |  |
| 41 | 80 | 7 | RLS | 1 |  |  |  |  |  |
| 41 | 80 | 12 | RLS | 2 |  |  |  |  |  |
| 41 | 60 | 10 | RLS | 1 |  |  |  |  |  |
| 41 | 60 | 5 | RLS | 2 |  |  |  |  |  |
| 43 | 80 | 0 | RLS | 1 |  |  |  |  |  |
| 43 | 80 | 0 | RLS | 2 |  |  |  |  |  |
| 43 | 60 | 0 | RLS | 1 |  |  |  |  |  |
| 43 | 60 | 0 | RLS | 2 |  |  |  |  |  |
| 44 | 80 | 0 | RLS+AUG | 1 |  |  |  |  |  |
| 44 | 80 | 0 | RLS+AUG | 2 |  |  |  |  |  |
| 44 | 60 | 1 | RLS+AUG | 1 |  |  |  |  |  |
| 44 | 60 | 0 | RLS+AUG | 2 |  |  |  |  |  |
| 45 | 80 | 0 | RLS+AUG | 1 |  |  |  |  |  |
| 45 | 80 | 0 | RLS+AUG | 2 |  |  |  |  |  |
| 45 | 60 | 0 | RLS+AUG | 1 |  |  |  |  |  |
| 45 | 60 | 0 | RLS+AUG | 2 |  |  |  |  |  |
| 46 | 80 | 0 | RLS | 1 |  |  |  |  |  |
| 46 | 80 | 0 | RLS | 2 |  |  |  |  |  |
| 46 | 60 | 2 | RLS | 1 |  |  |  |  |  |
| 46 | 60 | 3 | RLS | 2 |  |  |  |  |  |
| 47 | 80 | 0 | RLS+AUG | 1 |  |  |  |  |  |
| 47 | 80 | 0 | RLS+AUG | 2 |  |  |  |  |  |
| 47 | 60 | 2 | RLS+AUG | 1 |  |  |  |  |  |
| 47 | 60 | 3 | RLS+AUG | 2 |  |  |  |  |  |
| 48 | 80 | 1 | RLS | 1 |  |  |  |  |  |
| 48 | 80 | 0 | RLS | 2 |  |  |  |  |  |
| 48 | 60 | 0 | RLS | 1 |  |  |  |  |  |
| 48 | 60 | 0 | RLS | 2 |  |  |  |  |  |
| 50 | 80 | 3 | RLS | 1 |  |  |  |  |  |
| 50 | 80 | 5 | RLS | 2 |  |  |  |  |  |
| 50 | 60 | 8 | RLS | 1 |  |  |  |  |  |
| 50 | 60 | 10 | RLS | 2 |  |  |  |  |  |
| 51 | 80 | 0 | RLS+AUG | 1 |  |  |  |  |  |
| 51 | 80 | 0 | RLS+AUG | 2 |  |  |  |  |  |
| 51 | 60 | 0 | RLS+AUG | 1 |  |  |  |  |  |
| 51 | 60 | 0 | RLS+AUG | 2 |  |  |  |  |  |
| 52 | 80 | 0 | RLS | 1 |  |  |  |  |  |
| 52 | 80 | 0 | RLS | 2 |  |  |  |  |  |
| 52 | 60 | 0 | RLS | 1 |  |  |  |  |  |
| 52 | 60 | 0 | RLS | 2 |  |  |  |  |  |
| 53 | 80 | 5 | RLS+AUG | 1 |  |  |  |  |  |
| 53 | 80 | 6 | RLS+AUG | 2 |  |  |  |  |  |
| 53 | 60 | 12 | RLS+AUG | 1 |  |  |  |  |  |
| 53 | 60 | 17 | RLS+AUG | 2 |  |  |  |  |  |
| 54 | 80 | 0 | RLS | 1 |  |  |  |  |  |
| 54 | 80 | 0 | RLS | 2 |  |  |  |  |  |
| 54 | 60 | 0 | RLS | 1 |  |  |  |  |  |
| 54 | 60 | 0 | RLS | 2 |  |  |  |  |  |
| 55 | 80 | 0 | RLS+AUG | 1 |  |  |  |  |  |
| 55 | 80 | 0 | RLS+AUG | 2 |  |  |  |  |  |
| 55 | 60 | 0 | RLS+AUG | 1 |  |  |  |  |  |
| 55 | 60 | 2 | RLS+AUG | 2 |  |  |  |  |  |
| 56 | 80 | 0 | RLS | 1 |  |  |  |  |  |
| 56 | 80 | 0 | RLS | 2 |  |  |  |  |  |
| 56 | 60 | 0 | RLS | 1 |  |  |  |  |  |
| 56 | 60 | 0 | RLS | 2 |  |  |  |  |  |
| 58 | 80 | 0 | RLS+AUG | 1 |  |  |  |  |  |
| 58 | 80 | 3 | RLS+AUG | 2 |  |  |  |  |  |
| 58 | 60 | 19 | RLS+AUG | 1 |  |  |  |  |  |
| 58 | 60 | 24 | RLS+AUG | 2 |  |  |  |  |  |
| 62 | 80 | 0 | RLS+AUG | 1 |  |  |  |  |  |
| 62 | 80 | 0 | RLS+AUG | 2 |  |  |  |  |  |
| 62 | 60 | 0 | RLS+AUG | 1 |  |  |  |  |  |
| 62 | 60 | 0 | RLS+AUG | 2 |  |  |  |  |  |
| 63 | 80 | 5 | RLS+AUG | 1 |  |  |  |  |  |
| 63 | 80 | 5 | RLS+AUG | 2 |  |  |  |  |  |
| 63 | 60 | 7 | RLS+AUG | 1 |  |  |  |  |  |
| 63 | 60 | 4 | RLS+AUG | 2 |  |  |  |  |  |
| 64 | 80 | 5 | RLS | 1 |  |  |  |  |  |
| 64 | 80 | 3 | RLS | 2 |  |  |  |  |  |
| 64 | 60 | 5 | RLS | 1 |  |  |  |  |  |
| 64 | 60 | 7 | RLS | 2 |  |  |  |  |  |
| 65 | 80 | 0 | RLS+AUG | 1 |  |  |  |  |  |
| 65 | 80 | 0 | RLS+AUG | 2 |  |  |  |  |  |
| 65 | 60 | 1 | RLS+AUG | 1 |  |  |  |  |  |
| 65 | 60 | 0 | RLS+AUG | 2 |  |  |  |  |  |
| 66 | 80 | 0 | RLS+AUG | 1 |  |  |  |  |  |
| 66 | 80 | 0 | RLS+AUG | 2 |  |  |  |  |  |
| 66 | 60 | 0 | RLS+AUG | 1 |  |  |  |  |  |
| 66 | 60 | 0 | RLS+AUG | 2 |  |  |  |  |  |
| 69 | 80 | 2 | RLS+AUG | 1 |  |  |  |  |  |
| 69 | 80 | 0 | RLS+AUG | 2 |  |  |  |  |  |
| 69 | 60 | 4 | RLS+AUG | 1 |  |  |  |  |  |
| 69 | 60 | 1 | RLS+AUG | 2 |  |  |  |  |  |
| 70 | 60 | 4 | RLS+AUG | 1 |  |  |  |  |  |
| 70 | 80 | 0 | RLS+AUG | 2 |  |  |  |  |  |
| 70 | 60 | 0 | RLS+AUG | 1 |  |  |  |  |  |
| 70 | 80 | 0 | RLS+AUG | 2 |  |  |  |  |  |
| 72 | 80 | 0 | RLS+AUG | 1 |  |  |  |  |  |
| 72 | 80 | 0 | RLS+AUG | 2 |  |  |  |  |  |
| 72 | 60 | 0 | RLS+AUG | 1 |  |  |  |  |  |
| 72 | 60 | 1 | RLS+AUG | 2 |  |  |  |  |  |
| 73 | 80 | 0 | RLS+AUG | 1 |  |  |  |  |  |
| 73 | 80 | 0 | RLS+AUG | 2 |  |  |  |  |  |
| 73 | 60 | 0 | RLS+AUG | 1 |  |  |  |  |  |
| 73 | 60 | 0 | RLS+AUG | 2 |  |  |  |  |  |
| 74 | 80 | 1 | RLS+AUG | 1 |  |  |  |  |  |
| 74 | 80 | 1 | RLS+AUG | 2 |  |  |  |  |  |
| 74 | 60 | 7 | RLS+AUG | 1 |  |  |  |  |  |
| 74 | 60 | 6 | RLS+AUG | 2 |  |  |  |  |  |
| 75 | 80 | 6 | RLS+AUG | 1 |  |  |  |  |  |
| 75 | 80 | 8 | RLS+AUG | 2 |  |  |  |  |  |
| 75 | 60 | 15 | RLS+AUG | 1 |  |  |  |  |  |
| 75 | 60 | 13 | RLS+AUG | 2 |  |  |  |  |  |
| 76 | 80 | 0 | RLS+AUG | 1 |  |  |  |  |  |
| 76 | 80 | 1 | RLS+AUG | 2 |  |  |  |  |  |
| 76 | 60 | 0 | RLS+AUG | 1 |  |  |  |  |  |
| 76 | 60 | 1 | RLS+AUG | 2 |  |  |  |  |  |
| 77 | 80 | 0 | RLS+AUG | 1 |  |  |  |  |  |
| 77 | 80 | 0 | RLS+AUG | 2 |  |  |  |  |  |
| 77 | 60 | 0 | RLS+AUG | 1 |  |  |  |  |  |
| 77 | 60 | 0 | RLS+AUG | 2 |  |  |  |  |  |
| 78 | 80 | 4 | RLS+AUG | 1 |  |  |  |  |  |
| 78 | 80 | 10 | RLS+AUG | 2 |  |  |  |  |  |
| 78 | 60 | 12 | RLS+AUG | 1 |  |  |  |  |  |
| 78 | 60 | 9 | RLS+AUG | 2 |  |  |  |  |  |
| 79 | 80 | 4 | RLS | 1 |  |  |  |  |  |
| 79 | 80 | 5 | RLS | 2 |  |  |  |  |  |
| 79 | 60 | 6 | RLS | 1 |  |  |  |  |  |
| 79 | 60 | 4 | RLS | 2 |  |  |  |  |  |
| 84 | 80 | 0 | RLS+AUG | 1 |  |  |  |  |  |
| 84 | 80 | 0 | RLS+AUG | 2 |  |  |  |  |  |
| 84 | 60 | 0 | RLS+AUG | 1 |  |  |  |  |  |
| 84 | 60 | 0 | RLS+AUG | 2 |  |  |  |  |  |
| 85 | 80 | 0 | RLS | 1 |  |  |  |  |  |
| 85 | 80 | 0 | RLS | 2 |  |  |  |  |  |
| 85 | 60 | 0 | RLS | 1 |  |  |  |  |  |
| 85 | 60 | 0 | RLS | 2 |  |  |  |  |  |
| 107 | 80 | 1 | Controls | 1 |  |  |  |  |  |
| 107 | 80 | 1 | Controls | 2 |  |  |  |  |  |
| 107 | 60 | 0 | Controls | 1 |  |  |  |  |  |
| 107 | 60 | 0 | Controls | 2 |  |  |  |  |  |
| 108 | 80 | 3 | Controls | 1 |  |  |  |  |  |
| 108 | 80 | 4 | Controls | 2 |  |  |  |  |  |
| 108 | 60 | 7 | Controls | 1 |  |  |  |  |  |
| 108 | 60 | 5 | Controls | 2 |  |  |  |  |  |
| 109 | 80 | 6 | Controls | 1 |  |  |  |  |  |
| 109 | 80 | 3 | Controls | 2 |  |  |  |  |  |
| 109 | 60 | 12 | Controls | 1 |  |  |  |  |  |
| 109 | 60 | 9 | Controls | 2 |  |  |  |  |  |
| 110 | 80 | 6 | Controls | 1 |  |  |  |  |  |
| 110 | 80 | 8 | Controls | 2 |  |  |  |  |  |
| 110 | 60 | 15 | Controls | 1 |  |  |  |  |  |
| 110 | 60 | 10 | Controls | 2 |  |  |  |  |  |
| 111 | 80 | 1 | Controls | 1 |  |  |  |  |  |
| 111 | 80 | 1 | Controls | 2 |  |  |  |  |  |
| 111 | 60 | 5 | Controls | 1 |  |  |  |  |  |
| 111 | 60 | 3 | Controls | 2 |  |  |  |  |  |
| 112 | 80 | 0 | Controls | 1 |  |  |  |  |  |
| 112 | 80 | 0 | Controls | 2 |  |  |  |  |  |
| 112 | 60 | 0 | Controls | 1 |  |  |  |  |  |
| 112 | 60 | 0 | Controls | 2 |  |  |  |  |  |
| 113 | 80 | 0 | Controls | 1 |  |  |  |  |  |
| 113 | 80 | 3 | Controls | 2 |  |  |  |  |  |
| 113 | 60 | 9 | Controls | 1 |  |  |  |  |  |
| 113 | 60 | 10 | Controls | 2 |  |  |  |  |  |
| 114 | 80 | 5 | Controls | 1 |  |  |  |  |  |
| 114 | 80 | 3 | Controls | 2 |  |  |  |  |  |
| 114 | 60 | 4 | Controls | 1 |  |  |  |  |  |
| 114 | 60 | 3 | Controls | 2 |  |  |  |  |  |
| 115 | 80 | 3 | Controls | 1 |  |  |  |  |  |
| 115 | 80 | 4 | Controls | 2 |  |  |  |  |  |
| 115 | 60 | 5 | Controls | 1 |  |  |  |  |  |
| 115 | 60 | 8 | Controls | 2 |  |  |  |  |  |
| 116 | 80 | 4 | Controls | 1 |  |  |  |  |  |
| 116 | 80 | 6 | Controls | 2 |  |  |  |  |  |
| 116 | 60 | 16 | Controls | 1 |  |  |  |  |  |
| 116 | 60 | 8 | Controls | 2 |  |  |  |  |  |
| 117 | 80 | 5 | Controls | 1 |  |  |  |  |  |
| 117 | 80 | 5 | Controls | 2 |  |  |  |  |  |
| 117 | 60 | 26 | Controls | 1 |  |  |  |  |  |
| 117 | 60 | 26 | Controls | 2 |  |  |  |  |  |
| 118 | 80 | 5 | Controls | 1 |  |  |  |  |  |
| 118 | 80 | 4 | Controls | 2 |  |  |  |  |  |
| 118 | 60 | 11 | Controls | 1 |  |  |  |  |  |
| 118 | 60 | 11 | Controls | 2 |  |  |  |  |  |
| 119 | 80 | 2 | Controls | 1 |  |  |  |  |  |
| 119 | 80 | 3 | Controls | 2 |  |  |  |  |  |
| 119 | 60 | 1 | Controls | 1 |  |  |  |  |  |
| 119 | 60 | 4 | Controls | 2 |  |  |  |  |  |
| 120 | 80 | 5 | Controls | 1 |  |  |  |  |  |
| 120 | 80 | 5 | Controls | 2 |  |  |  |  |  |
| 120 | 60 | 10 | Controls | 1 |  |  |  |  |  |
| 120 | 60 | 13 | Controls | 2 |  |  |  |  |  |
| 121 | 80 | 5 | Controls | 1 |  |  |  |  |  |
| 121 | 80 | 6 | Controls | 2 |  |  |  |  |  |
| 121 | 60 | 6 | Controls | 1 |  |  |  |  |  |
| 121 | 60 | 5 | Controls | 2 |  |  |  |  |  |
| 122 | 80 | 8 | Controls | 1 |  |  |  |  |  |
| 122 | 80 | 11 | Controls | 2 |  |  |  |  |  |
| 122 | 60 | 9 | Controls | 1 |  |  |  |  |  |
| 122 | 60 | 8 | Controls | 2 |  |  |  |  |  |
| 123 | 80 | 0 | Controls | 1 |  |  |  |  |  |
| 123 | 80 | 0 | Controls | 2 |  |  |  |  |  |
| 123 | 60 | 0 | Controls | 1 |  |  |  |  |  |
| 123 | 60 | 0 | Controls | 2 |  |  |  |  |  |
| 124 | 80 | 6 | Controls | 1 |  |  |  |  |  |
| 124 | 80 | 3 | Controls | 2 |  |  |  |  |  |
| 124 | 60 | 7 | Controls | 1 |  |  |  |  |  |
| 124 | 60 | 5 | Controls | 2 |  |  |  |  |  |
| 125 | 80 | 0 | Controls | 1 |  |  |  |  |  |
| 125 | 80 | 0 | Controls | 2 |  |  |  |  |  |
| 125 | 60 | 0 | Controls | 1 |  |  |  |  |  |
| 125 | 60 | 0 | Controls | 2 |  |  |  |  |  |
| 126 | 80 | 6 | Controls | 1 |  |  |  |  |  |
| 126 | 80 | 8 | Controls | 2 |  |  |  |  |  |
| 126 | 60 | 12 | Controls | 1 |  |  |  |  |  |
| 126 | 60 | 9 | Controls | 2 |  |  |  |  |  |
| 127 | 80 | 5 | Controls | 1 |  |  |  |  |  |
| 127 | 80 | 8 | Controls | 2 |  |  |  |  |  |
| 127 | 60 | 12 | Controls | 1 |  |  |  |  |  |
| 127 | 60 | 14 | Controls | 2 |  |  |  |  |  |
|  |  |  |  |  |  |  |  |  |  |
| **SUBJECT** | **GROUP** | **OPPOSITE COLOUR CHOICE** |  |  |  |  |  |  |  |
| 1 | RLS-AUG | 2 |  |  |  |  |  |  |  |
| 2 | RLS-AUG | 0 |  |  |  |  |  |  |  |
| 3 | RLS-AUG | 0 |  |  |  |  |  |  |  |
| 4 | RLS+AUG | 4 |  |  |  |  |  |  |  |
| 5 | RLS+AUG | 3 |  |  |  |  |  |  |  |
| 6 | RLS+AUG | 0 |  |  |  |  |  |  |  |
| 8 | RLS+AUG | 0 |  |  |  |  |  |  |  |
| 9 | RLS+AUG | 0 |  |  |  |  |  |  |  |
| 10 | RLS+AUG | 0 |  |  |  |  |  |  |  |
| 11 | RLS+AUG | 0 |  |  |  |  |  |  |  |
| 12 | RLS+AUG | 3 |  |  |  |  |  |  |  |
| 14 | RLS+AUG | 2 |  |  |  |  |  |  |  |
| 15 | RLS+AUG | 1 |  |  |  |  |  |  |  |
| 16 | RLS-AUG | 2 |  |  |  |  |  |  |  |
| 17 | RLS+AUG | 3 |  |  |  |  |  |  |  |
| 18 | RLS-AUG | 0 |  |  |  |  |  |  |  |
| 20 | RLS+AUG | 2 |  |  |  |  |  |  |  |
| 22 | RLS-AUG | 3 |  |  |  |  |  |  |  |
| 23 | RLS-AUG | 2 |  |  |  |  |  |  |  |
| 24 | RLS+AUG | 3 |  |  |  |  |  |  |  |
| 26 | RLS-AUG | 5 |  |  |  |  |  |  |  |
| 27 | RLS+AUG | 2 |  |  |  |  |  |  |  |
| 30 | RLS-AUG | 1 |  |  |  |  |  |  |  |
| 31 | RLS-AUG | 0 |  |  |  |  |  |  |  |
| 32 | RLS+AUG | 2 |  |  |  |  |  |  |  |
| 34 | RLS+AUG | 1 |  |  |  |  |  |  |  |
| 35 | RLS-AUG | 0 |  |  |  |  |  |  |  |
| 36 | RLS+AUG | 2 |  |  |  |  |  |  |  |
| 37 | RLS+AUG | 0 |  |  |  |  |  |  |  |
| 38 | RLS-AUG | 2 |  |  |  |  |  |  |  |
| 39 | RLS-AUG | 2 |  |  |  |  |  |  |  |
| 40 | RLS+AUG | 2 |  |  |  |  |  |  |  |
| 41 | RLS-AUG | 0 |  |  |  |  |  |  |  |
| 43 | RLS-AUG | 0 |  |  |  |  |  |  |  |
| 44 | RLS+AUG | 1 |  |  |  |  |  |  |  |
| 45 | RLS+AUG | 8 |  |  |  |  |  |  |  |
| 46 | RLS-AUG | 0 |  |  |  |  |  |  |  |
| 47 | RLS+AUG | 1 |  |  |  |  |  |  |  |
| 48 | RLS-AUG | 4 |  |  |  |  |  |  |  |
| 50 | RLS-AUG | 0 |  |  |  |  |  |  |  |
| 51 | RLS+AUG | 6 |  |  |  |  |  |  |  |
| 52 | RLS-AUG | 3 |  |  |  |  |  |  |  |
| 53 | RLS+AUG | 3 |  |  |  |  |  |  |  |
| 54 | RLS-AUG | 1 |  |  |  |  |  |  |  |
| 55 | RLS+AUG | 0 |  |  |  |  |  |  |  |
| 56 | RLS-AUG | 0 |  |  |  |  |  |  |  |
| 58 | RLS+AUG | 0 |  |  |  |  |  |  |  |
| 62 | RLS+AUG | 4 |  |  |  |  |  |  |  |
| 63 | RLS+AUG | 0 |  |  |  |  |  |  |  |
| 64 | RLS-AUG | 0 |  |  |  |  |  |  |  |
| 65 | RLS+AUG | 1 |  |  |  |  |  |  |  |
| 66 | RLS+AUG | 1 |  |  |  |  |  |  |  |
| 69 | RLS+AUG | 0 |  |  |  |  |  |  |  |
| 70 | RLS+AUG | 2 |  |  |  |  |  |  |  |
| 72 | RLS+AUG | 1 |  |  |  |  |  |  |  |
| 73 | RLS+AUG | 6 |  |  |  |  |  |  |  |
| 74 | RLS+AUG | 5 |  |  |  |  |  |  |  |
| 75 | RLS+AUG | 0 |  |  |  |  |  |  |  |
| 76 | RLS+AUG | 1 |  |  |  |  |  |  |  |
| 77 | RLS+AUG | 1 |  |  |  |  |  |  |  |
| 78 | RLS+AUG | 0 |  |  |  |  |  |  |  |
| 79 | RLS-AUG | 0 |  |  |  |  |  |  |  |
| 84 | RLS+AUG | 9 |  |  |  |  |  |  |  |
| 85 | RLS-AUG | 2 |  |  |  |  |  |  |  |
| 107 | Control | 1 |  |  |  |  |  |  |  |
| 108 | Control | 0 |  |  |  |  |  |  |  |
| 109 | Control | 0 |  |  |  |  |  |  |  |
| 110 | Control | 0 |  |  |  |  |  |  |  |
| 111 | Control | 0 |  |  |  |  |  |  |  |
| 112 | Control | 1 |  |  |  |  |  |  |  |
| 113 | Control | 0 |  |  |  |  |  |  |  |
| 114 | Control | 0 |  |  |  |  |  |  |  |
| 115 | Control | 0 |  |  |  |  |  |  |  |
| 116 | Control | 0 |  |  |  |  |  |  |  |
| 117 | Control | 0 |  |  |  |  |  |  |  |
| 118 | Control | 0 |  |  |  |  |  |  |  |
| 119 | Control | 2 |  |  |  |  |  |  |  |
| 120 | Control | 0 |  |  |  |  |  |  |  |
| 121 | Control | 0 |  |  |  |  |  |  |  |
| 122 | Control | 0 |  |  |  |  |  |  |  |
| 123 | Control | 3 |  |  |  |  |  |  |  |
| 124 | Control | 0 |  |  |  |  |  |  |  |
| 125 | Control | 0 |  |  |  |  |  |  |  |
| 126 | Control | 0 |  |  |  |  |  |  |  |
| 127 | Control | 0 |  |  |  |  |  |  |  |
